# Visual preference of plant features in different living environments using eye tracking and EEG

**DOI:** 10.1371/journal.pone.0279596

**Published:** 2022-12-30

**Authors:** Ningning Ding, Yongde Zhong, Jiaxiang Li, Qiong Xiao, Shuangquan Zhang, Hongling Xia

**Affiliations:** 1 Central South University of Forestry and Technology, Changsha, China; 2 National Forestry and Grassland Administration State Forestry Administration Engineering Research Center for Forest Tourism, Changsha, China; 3 Hunan Urban Construction College, Xiangtan, China; Universiti Tunku Abdul Rahman, MALAYSIA

## Abstract

Plants play a very important role in landscape construction. In order to explore whether different living environment will affect people’s preference for the structural features of plant organs, this study examined 26 villagers and 33 college students as the participants, and pictures of leaves, flowers and fruits of plants as the stimulus to conduct eye-tracking and EEG detection experiments. We found that eye movement indicators can explain people’s visual preferences, but they are unable to find differences in preferences between groups. EEG indicators can make up for this deficiency, which further reveals the difference in psychological and physiological responses between the two groups when viewing stimuli. The final results show that the villagers and the students liked leaves best, preferring aciculiform and leathery leaves; solitary, purple and capitulum flowers; and medium-sized, spathulate, black and pear fruits. In addition, it was found that the overall attention of the villagers when watching stimuli was far lower than that of the students, but the degree of meditation was higher. With regard to eye movement and EEG, the total duration of fixations is highly positively correlated with the number of fixations, and the average pupil size has a weak negative correlation with attention. On the contrary, the average duration of fixations has a weak positive correlation with meditation. Generally speaking, we believe that *Photinia×fraseri*, *Metasequoia glyptostroboides*, *Photinia serratifolia*, *Koelreuteria bipinnata* and *Cunninghamia lanceolata* are superior landscape building plants in rural areas and on campuses; *Pinus thunbergii*, *Myrica rubra*, *Camellia japonica* and other plants with obvious features and bright colours are also the first choice in rural landscapes; and *Yulania biondii*, *Cercis chinensis*, *Hibiscus mutabilis* and other plants with simple structures are the first choice in campus landscapes. This study is of great significance for selecting plants for landscape construction and management according to different environments and local conditions.

## Introduction

Environmental quality plays an important role in our life, which affects appeal to people’s aesthetic sense, and their physiological and psychological health [1]. People can perceive and evaluate the environment through sight, hearing, smell and touch, but visual perception is the most important means [2]. Some scholars use the term “visual environment” to describe people’s visual perception of the environment [3, 4], and many existing studies also use the term “visual landscape” to highlight the visual attributes of landscape [2, 5]. The visual preference of the landscape means the environment is conveyed by visual perception, and people form inner perceptual feelings based on personal experience, needs, expectations and psychological state [6, 7]. The range of landscape is very wide, encompassing everything from the macro- to the micro-scale, from urban parks to plants [8]; plant organs can also be called environment.

Some scholars have focused on the landscape and explored people’s preference for different landscape types or features. They have selected cities [9], parks [10], courtyards [11], green spaces [12] and the river bank [13], among others, on as the research objects, and summarized nine visual concepts [14] used to analyze the visual landscape features: naturalness, interference, complexity, consistency, historicity, management work, horizon, image and time variation. In addition to the above landscape types, increasing numbers of scholars have done much meaningful research on plant landscape preference. Plants are an important element in the landscape, with the capacity to improve people’s mental and physical health. Any vegetation contributes to visual improvement [15], and is also an important element of environmental perception and preference [16], so it cannot be ignored. People often start from individual plants [15], communities or plant configuration combinations [17], and study them from a macro perspective. The structural features and systems of plants have an important influence on how people interpret plants [18], so there are also some researchers who cut in from a deeper direction to study the public’s preference for plant organs. Rahnema et al. [19] selected eight ornamental plants with flowers or leaves, and used questionnaires to reveal the preference and emotional perception of ornamental plants from the perspective of plant organ features. Tarakci-Eren and Duzenli [16] took photos of designated angiosperms in different seasons, and studied the preference level and cognitive differences of the colour changes of stems, leaves, flowers and fruits of plants on the Karadeniz Technical University campus. However, it is worth noting that these scholars have not undertaken this study of plant organs as the core of their research, so they have not systematically integrated people’s preferences when organs are used as plant landscapes.

As early as several decades ago, scholars proposed that most people viewing a landscape would determine the advantages and disadvantages of the landscape [20], and thought that different people would have different views when viewing the same landscape [21, 22]. Therefore, in addition to the above-mentioned research on landscape preferences, some scholars pay attention to the differences in the preferences of different individuals or groups for particular landscapes. Most are based on age and gender [23], cultural background [24], professional background [25] and familiarity with the research location [26, 27] as individual or group features. In addition, landscape preference is not only influenced by people’s natural self-awareness [28], but also impacted by the growing environment to a certain extent, so the living environment is also a very important group characteristic. Yet there are currently few research results. The differences in living environment span a wide range and can exist in different countries. Some scholars have investigated the differences in preferences of people who live in different countries and have different living environments for urban square design elements [29]. There may also be differences in living environment within the same country. For example, Luo et al. [30] took China as an example and compared local high school students in Xiamen and Xinjiang. Through the implementation of landscape livability and aesthetic preference tests, the landscape preference differences and their features and the assimilation trend between the two groups of people were discussed. In some studies, the concept of “prototype landscape” was used to distinguish people from different living environments. This concept is the experience summary of settlers’ previous living landscapes [31]; Gong et al. [32] discuss the influence of different original landscape types on the perception of the urban plant landscape.

The rural residential environment is an important part of livable and sustainable rural areas all over the world. Planting trees can change the perception of landscape, and different tree configurations will produce different responses [33]. With the development of social technology and the changing rural economy, the configuration of the rural landscape is being redrawn [34]. A campus is a miniature of the general trend of urban forest development. The College of Botany, Landscape Architecture and Urban Design of The University of Pennsylvania in Philadelphia in the United States once made a comprehensive landscape plan for its campus, and advocated the planting and cultivation of campus trees [35]. Hami and Abdi [36] also emphasize the importance of vegetation, rest areas and waterscape for a campus landscape, because students like campuses with natural foundations, landscape elements and places where they can sit and rest. Study areas should be designed with vertical natural elements. However, the best plants for landscape construction in the two environments have not been studied extensively. We therefore decided to explore whether differences exist in people’s preferences for particular plant landscapes in rural and campus living environments. Villagers and students are the biggest landscape audiences in rural and campus environments respectively. They spend most of their time in rural and campus environments, making them the best candidates to study in relation to the two environments; we therefore chose to make villagers and students the research objects of this study.

People’s attitudes, perceptions and preferences are closely related to the features of eye movement, and people’s cognitive behavior can be analyzed using eye movement [37]. Eye movement is the movement of the eyes when they receive external information, which mainly includes three basic forms: fixation, saccade and pursuit movement [38]. Among these, fixation refers to aiming the central fossa of the eyeball at the stimulate target in order to get the clearest image. However, the eyeball is not completely stationary when staring, and there are still very small saccade movements, such as drift, tremor and involuntary saccade. The saccade is the extremely fast movement of the fixation point, thus keeping the object to be fixed in the central fovea area, in order to search for the stimulation target and obtain clear vision. Pursuit movement refers to the movement of people’s eyes following a moving object [39]. People usually think they control eye movements, but in fact there are many eye movements that are difficult for individuals to control or even detect. The above three kinds of eye movements always exist in people’s daily life consciously or unconsciously. According to statistics, 80 to 90 per cent of the information people get from the outside world comes from vision [40], so eye tracking has wide application value in many fields [41–45]. This method has gradually matured and is widely used in the study of visual landscape preference, especially in the forest [46, 47], urban parks [10, 48] and rural landscapes [49]. As mentioned above, however, few studies exist on visual preference using eye tracking experiments for individual plants and their organs at a closer distance. For example, Zheng et al. [18] used the pictures of ornamental bamboo stems and leaves as their stimulus materials for eye tracking experiments, and discussed people’s preference types from stem colours to stem stripes, leaf stripes, stem variation types and growth habits.

In addition to eye tracking, EEG technology has also been applied to landscape evaluation and preference. The development of neuroscience over the last century has greatly enriched people’s understanding of the bioelectrical signals emitted by brain neurons [50]. EEG is a physiological signal that uses physiological methods to measure nerve signals on the surface of the scalp and record them. The patterns and frequencies of these bioelectric signals can be measured by sensors placed on the scalp [51]. Different neural activities produce different brain wave patterns, and different brain wave patterns emit EEG with different frequencies, thus showing different brain states. The following are the frequency band divisions and the mental state of the brain reflected by different types of EEG:

1. Delta wave: 1–3 Hz, in deep sleep, non-rapid eye sleep, unconscious state
2. Theta wave: 4–7 Hz, in the state of creation, memory, fantasy, imagination, light sleep state
3. Alpha wave: 8–12 Hz, in a relaxed but not sleepy, calm and conscious state
4. Low-frequency Beta wave: 12–15 Hz, in a motor-sensory rhythm—that is, a state of relaxation, concentration and coordination
5. Intermediate-frequency Beta wave: 16–20 Hz, in a state of thinking and clear awareness of self and surrounding environment
6. High-frequency Beta wave: 21–30 Hz, in a state of alertness and excitement.

Many different psychological indicators can be obtained using this technique, which may infer attention from the electrical signals of the brain [41]. EEG response reveals the difference of electrical activity in the human brain [52]. The application of EEG mostly focuses on clinical diagnosis and research in medicine [53, 54]. In recent years, scholars have also applied this technology to other fields, such as driving a motor vehicle [55, 56], click intention of network users [57], consumer preference [58, 59] and landscape evaluation [60, 61], but the influence of plants on psychophysiological response has not been explored thoroughly [62]. When combined with other instruments, EEG can provide more accurate results for participants’ response under stimulation [63, 64]. Therefore, some researchers have combined eye tracking and EEG technology to discover people’s preferences and physiological responses to the landscape more scientifically and effectively [51, 65]. On the whole, however, the number of related studies remains small.

This study combines visual and neural science technologies–eye tracking and EEG–to study the preferences and differences of plant features between villagers and students living in different environments to provide a basis for a discussion of the similarities and differences of plant application in rural and campus landscape construction. We take plant organs as our research core, choose leaves, flowers and fruits as plant landscapes, and extract their colour, shape, size, type and other characteristics for research into preferences. The aims were to provide scientific reference suggestions for the selection of plants in landscape construction, explore some new perspectives for landscape preference, and provide more reference data for the combination of eye movement and EEG and the relationship between them.

## Methods

### Participants

There are two groups of participants in this experiment. One group comprises 26 villagers (M_age_ = 47.23, SD_age_ = 10.359) in Changkou Village, Sanming City, Fujian Province, with education levels of high school and below. They have lived in Changkou Village for at least two years and represent the group living in the rural environment. The other group consists of 33 undergraduates and postgraduates (M_age_ = 21.79, SD_age_ = 2.176) in Central South University of Forestry and Technology, Changsha City, Hunan Province. They are sophomores and above–that is, they have lived in the university for at least one year, representing the group living in the campus environment. The villagers in Changkou Village were organized to participate in the experiment by the staff of the village committee with the consent of the participants themselves, while the students in the university were recruited by the researchers through social media software on WeChat to publish recruitment information in the university via group chats. A total of 59 people participated in the experiment, including 20 men and 39 women, aged between 18 and 63 years. All participants had normal or corrected-to-normal vision, no colour blindness or weak colour recognition, and all of them passed the eye movement calibration in the experiment, meeting the experimental requirements. The experiment obtained the informed consent of all subjects. The study was carried out in accordance with relevant guidelines and regulations, and approved by the academic committee of Central South University of Forestry and Technology. The data were analyzed anonymously.

### Stimuli

**Fig 1:**
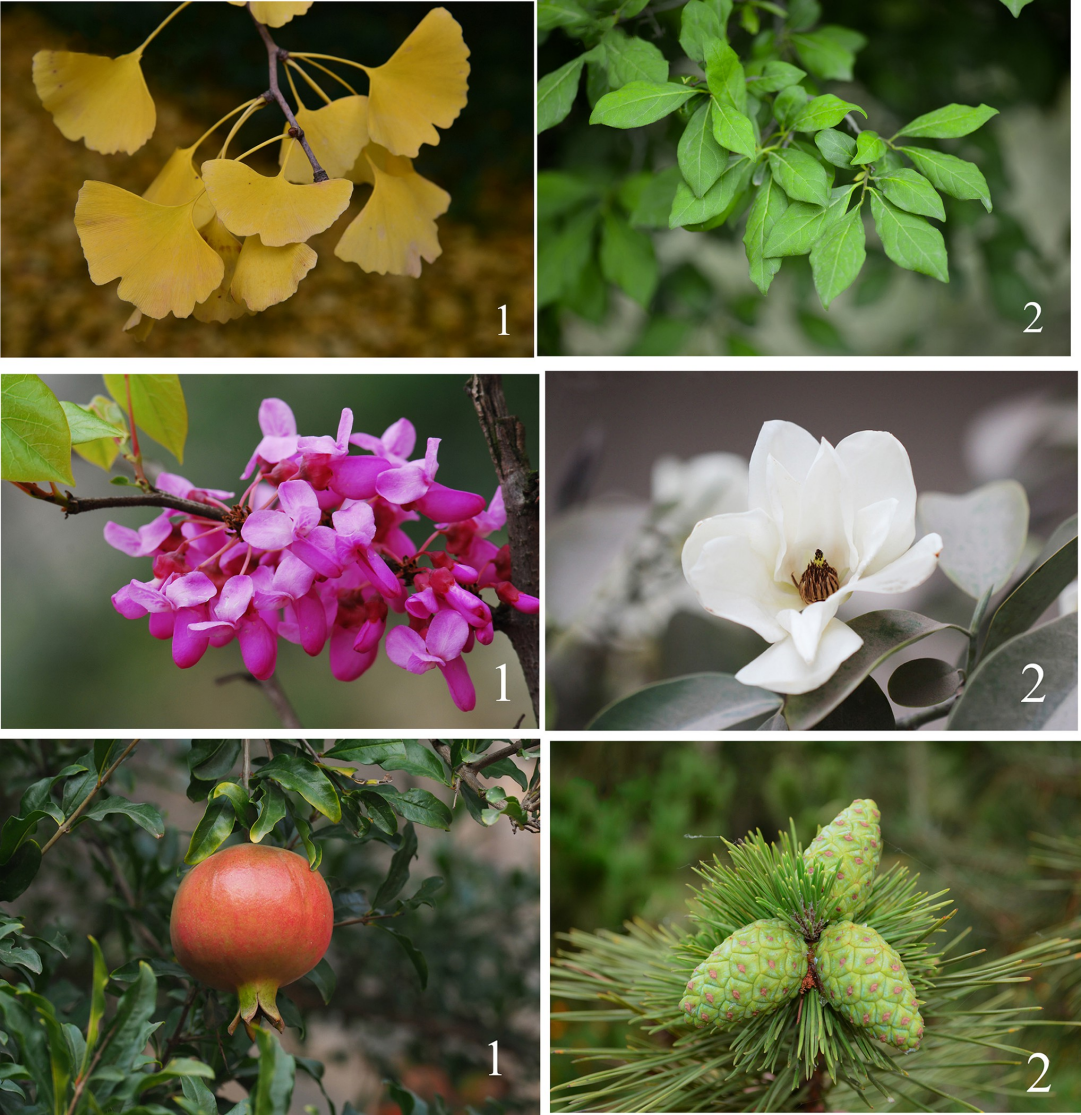
Pictures of some plant organs used in experiments. (a) leaves. 1 is the leaves of *Ginkgo biloba*, which is sector, yellow and leathery; 2 is the leaves of *Diospyros rhombifolia*, which is oval, green and paper-like. (b) Flowers. 1 is the flower of *Cercis chinensis*, with is raceme and purple; 2 is the flower of *Michelia maudiae*, which is solitary and white. (c) Fruits. 1 is the fruit of *Punica granatum*, which is spherical, berry, red and large; 2 is the fruit of *Pinus thunbergii*, which is oval, cone, green and medium.

It is considered effective to experiment with pictures as a medium [66–68]. The participants need to watch a total of 123 colour pictures, of which three are warm-up pictures set to prevent interference of the primary effect. The three warm-up pictures are first shown to the participants in the experiment, but their data are not included in any statistical analysis, and the remaining 120 are experimental pictures to obtain valid experimental data. Each picture uses depth-of-field mode of camera or fuzzy processing to ensure that one of the leaves, flowers or fruits of a certain plant can be clearly and intensively displayed. These pictures reflect some specific attributes of various plant organs, such as colour, shape, inflorescence, size, and so on (Fig 1). For the sake of explaining the size of fruit, this study called fruit with a diameter greater than 5 cm “large fruit”, that with a diameter of 3–5 centimetres “medium fruit”, that with a diameter of 1–3 centimetres “small fruit” and that with a diameter of less than 1 centimeter “micro fruit”. There are 52 species and 25 families of plants involved in the experiment, which were selected under the guidance of botanical experts (Table 1). Images were taken by researchers or downloaded from official websites related to plants, including the Institute of Botany, Chinese Academy of Sciences (http://www.ibcas.ac.cn), and Chinese Union of Botanical Gardens (https://www.cubg.cn). In order to reduce the interference of different tones on vision, Photoshop CS6 software was used to unify the tones of the pictures without changing the colours of plants.

**Table 1:** The species, families and numbers of plants used in this study.

Number of family	Family	Number of species	Species
1	*Cupressaceae*	6	*Fokienia hodginsii*
		50	*Metasequoia glyptostroboides*
		51	*Cunninghamia lanceolata*
2	*Cannabaceae*	20	*Celtis sinensis*
3	*Aquifoliaceae*	29	*Ilex rotunda*
4	*Fabaceae*	38	*Cercis chinensis*
5	*Elaeocarpaceae*	30	*Elaeocarpus glabripetalus*
6	*Pittosporaceae*	8	*Pittosporum tobira*
7	*Juglandaceae*	5	*Pterocarya stenoptera*
8	*Buxaceae*	12	*Buxus sinica*
9	*Hamamelidaceae*	4	*Liquidambar formosana*
		31	*Distylium racemosum*
		43	*Loropetalum chinense*
10	*Malvaceae*	45	*Hibiscus mutabilis*
11	*Calycanthaceae*	13	*Chimonanthus praecox*
12	*Magnoliaceae*	3	*Yulania liliiflora*
		9	*Michelia chapensis*
		15	*Yulania biondii*
		23	*Michelia maudiae*
		42	*Magnolia grandiflora*
		47	*Liriodendron chinense*
		49	*Michelia figo*
13	*Oleaceae*	11	*Fraxinus hubeiensis*
		16	*Ligustrum lucidum*
		19	*Osmanthus fragrans*
		48	*Jasminum mesnyi*
14	*Anacardiaceae*	17	*Choerospondias axillaris*
15	*Lythraceae*	25	*Punica granatum*
		39	*Lagerstroemia indica*
16	*Rosaceae*	2	*Cerasus campanulata*
		26	*Malus halliana*
		27	*Amygdalus persica*
		36	*Chaenomeles speciosa*
		37	*Photinia×fraseri*
		52	*Photinia serratifolia*
17	*Theaceae*	34	*Camellia sasanqua*
		41	*Camellia transarisanensis*
		46	*Camellia japonica*
18	*Ebenaceae*	14	*Diospyros rhombifolia*
19	*Pinaceae*	10	*Pinus thunbergii*
		44	*Pinus massoniana*
20	*Sapindaceae*	7	*Koelreuteria bipinnata*
		22	*Acer buergerianum*
		32	*Sapindus saponaria*
21	*Araliaceae*	1	*Fatsia japonica*
22	*Berberidaceae*	18	*Berberis thunbergii*
		21	*Nandina domestica*
		24	*Mahonia fortunei*
23	*Myricaceae*	40	*Myrica rubra*
24	*Ginkgoaceae*	33	*Ginkgo biloba*
25	*Lauraceae*	28	*Cinnamomum japonicum*
		35	*Fokienia hodginsii*

### Apparatus

The eye movement data are recorded by Tobii Pro Glasses 2 wearable eye tracker, which has a wireless real-time observation function; its super wide-angle scene camera ensures that we can investigate the natural visual observation behavior. The eye tracker uses pupil corneal reflection and binocular dark pupil acquisition technology to track the eye movement of the participant, and supports slip compensation–that is, the data error compensation when the capitulum-mounted module moves slightly during the test–so the data quality will not be affected. With the slip compensation technology and the sampling rate of 100Hz (four eye-tracking cameras, each with a sampling rate of 100Hz), more accurate data will be obtained. EEG data are collected by Mind Wave Mobile capitulumset equipment of the NeuroSky Company, which is connected to smartphone through Bluetooth. EEG data collected by equipment are received and recorded by eegID software, with a recording frequency of once every second, and output in CSV format file. The equipment uses ThinkGear technology to amplify the original EEG signal and filter out the interference caused by environmental noise and pulse and muscle movement, to ensure a clear EEG signal can be collected. It measures EEG signals through a sensor placed on the forehead and a reference electrode contact placed on the ear, and obtains δ, θ, α, β, and γ signal data, which are processed by an integrated chip. It then calculates people’s current mental state in a digital exponential way by using the eSenseblem algorithm, and obtains the quantized eSense indicators value, including attention and meditation. The specific calculation formula is as follows:

Pa=(mγ+nβ+tα)×100

Pm=(xθ+yδ+zα)×100

Where *Pa* is attention; *γ*, *β*, and *α* are, respectively, the percentages of *γ* wave, *β* wave, and *α* wave in EEG energy; and *m*, *n*, and *t* are the weight coefficients of *γ* wave, *β* wave, and *α* wave, respectively;

where *Pm* is meditation; *θ*, *δ*, and *α* are, respectively, the percentage of *θ* wave, *δ* wave, and *α* wave in the energy of the EEG signal; and *x*, *y*, and *z* represent the weight coefficients of *θ* wave, *δ* wave, and *α* wave, respectively.

### Methodology

Experiments were carried out in the laboratory of the Tourism College of Central South University of Forestry and Technology and in the meeting room of Changkou Village Committee. To avoid inaccurate eye tracking and EEG acquisition caused by the participants’ nervousness due to their unfamiliarity with laboratory instruments and the environment, and the influence of external factors, before the experiment the participants sat in a comfortable chair and wore the eye tracker and EEG detector at the same time under the guidance of researchers. They then adapted and rested for a certain period of time until the discomfort was eliminated. In addition, the experimental environment was controlled to be quiet, dimly lit with suitable for temperature and humidity [69]. In the experiment, eye movement calibration was carried out first, with the researcher instructing the participants to hold eye movement calibration cards for calibration; the experiment was carried out only after the calibration was completed. The experimental pictures were displayed in random order, and each picture was played for 10 seconds, so the participants only needed to watch freely according to their daily viewing habits [49]. The participants did not know the specific purpose of the experiment [70], and the experiment process was lengthy, allowing the participants to pause at any time and rest for five to 10 seconds before continuing [22, 71]. After the experiment, the participants need to fill in their personal details forms, and the researcher gave each participant a red envelope containing cash to express gratitude.

### Indicator selection

After the experiment, a large amount of eye movement and EEG related data were obtained, and the indicator data suitable for this study needed to be selected according to the research needs. In terms of eye movement, taking into account the consistent viewing time of each image and the clearly defined area of interest (AOI), five eye movement indicators were selected in this study, including total duration of fixations (TDF), number of fixations (NF), average duration of fixations (ADF), average pupil size (APS) and average amplitude of saccades (AAS). Among them, total duration of fixations and number of fixations can be regarded as a measure of attractiveness [72, 73] and can reflect the visual preference of participants [74]. The greater the attraction, the greater the value of the two indicators. The average duration of fixations conveys to researchers the time taken for the participants to acquire and process the information displayed by the stimulus. The larger the average of fixations, the greater the information conveyed by the stimulus, and the more time needed by participants to understand [75, 76]. The average pupil size reflects the visual sensitivity of participants to stimuli [18]. The average amplitude of saccades reflects the range of information obtained. The larger the amplitude, the clearer the picture features, meaning the participants could directly reach the target area [77].

In terms of EEG, two indicators of attention and meditation were selected. The attention indicators show the intensity of participants’ mental concentration, and mental states such as distraction, trance, inattention and anxiety will reduce the attention indicators [78]. Meditation indicates the mental relaxation degree of the participants, and there is a clear correlation between increased levels of meditation and decreased brain activity. Mental states such as anxiety, agitation and sensory stimulation will reduce the meditation value [78].

### Data analysis

After the experiment, the data of participants with low eye tracking sampling rate were deleted, and 24 valid data of villagers and 30 valid data of students were obtained, totalling 54. By deleting the data with incomplete EEG data records or too many pauses, 17 valid data of villagers and 25 valid data of students were obtained, totalling 42. The eye movement data were processed in Tobii Pro Lab software based on leaves, flowers and fruits as AOI, and then the required eye movement data was exported. For EEG data, first the data of the first 30s was deleted when watching the warm up pictures, then the data of each experimental picture for 10s was intercepted, and the average value of all tested data per second was calculated for subsequent analysis. SPSS 20.0 was used for statistical analysis, nonparametric testing and correlation analysis.

## Results

### Eye movement data

#### Organs, species and families

According to the results of the Mann-Whitney U-test, on the whole there are significant differences between villagers and students in five eye movement indicators respectively (p<0.05). The students’ total duration of fixations, number of fixations, average pupil size and average amplitude of saccades is much higher than those of villagers, but the average duration of fixations is the reverse (Table 2). Multivariate analysis of variance showed that there was no significant interaction between different environmental background and plant organs and their features in eye movement indicators (p>0.05).

**Table 2:** Mean value of eye movement indicators of villagers and students as a whole.

Varieties	TDF		NF		ADF		APS		AAS	
	Mean	SD	Mean	SD	Mean	SD	Mean	SD	Mean	SD
Villagers	3.872	2.442	11.32	7.519	0.371	0.348	3.950	0.990	4.250	2.020
Students	5.109	2.517	16.89	8.267	0.321	0.181	4.264	0.725	5.425	2.119

TDF: Total duration of fixations; NF: Number of fixations; ADF: Average duration of fixations; APS: Average pupil size; AAS: Average amplitude of saccades.

With respect to plant organs, the eye movement indicators of villagers and students have the same trend, and Kruskal-Wallis H-test results show that there are significant differences in their total duration of fixations, number of fixations and average duration of fixations (p<0.05), but no differences in their average pupil size or average amplitude of saccades. Both groups had the longest total duration of fixations and the biggest number of fixations when watching leaves, followed by flowers and fruits, while the order of average duration of fixations was completely opposite, and fruits had the longest. On the whole, all the indicators of students are much higher than those of villagers except for the average duration of fixations (Table 3).

**Table 3:** Mean results of eye movement indicators of two populations on different plant organs.

Groups	Varieties	TDF	NF	ADF	APS	AAS
Villagers	Leaf	4.743	14.837	0.347	3.958	4.328
	Flower	3.715	10.400	0.381	3.941	4.215
	Fruit	3.152	8.710	0.384	3.950	4.207
Students	Leaf	6.091	21.300	0.303	4.296	5.452
	Flower	5.057	16.310	0.329	4.229	5.516
	Fruit	4.179	13.050	0.330	4.268	5.305

TDF: Total duration of fixations; NF: Number of fixations; ADF: Average duration of fixations; APS: Average pupil size; AAS: Average amplitude of saccades.

**Fig 2:**
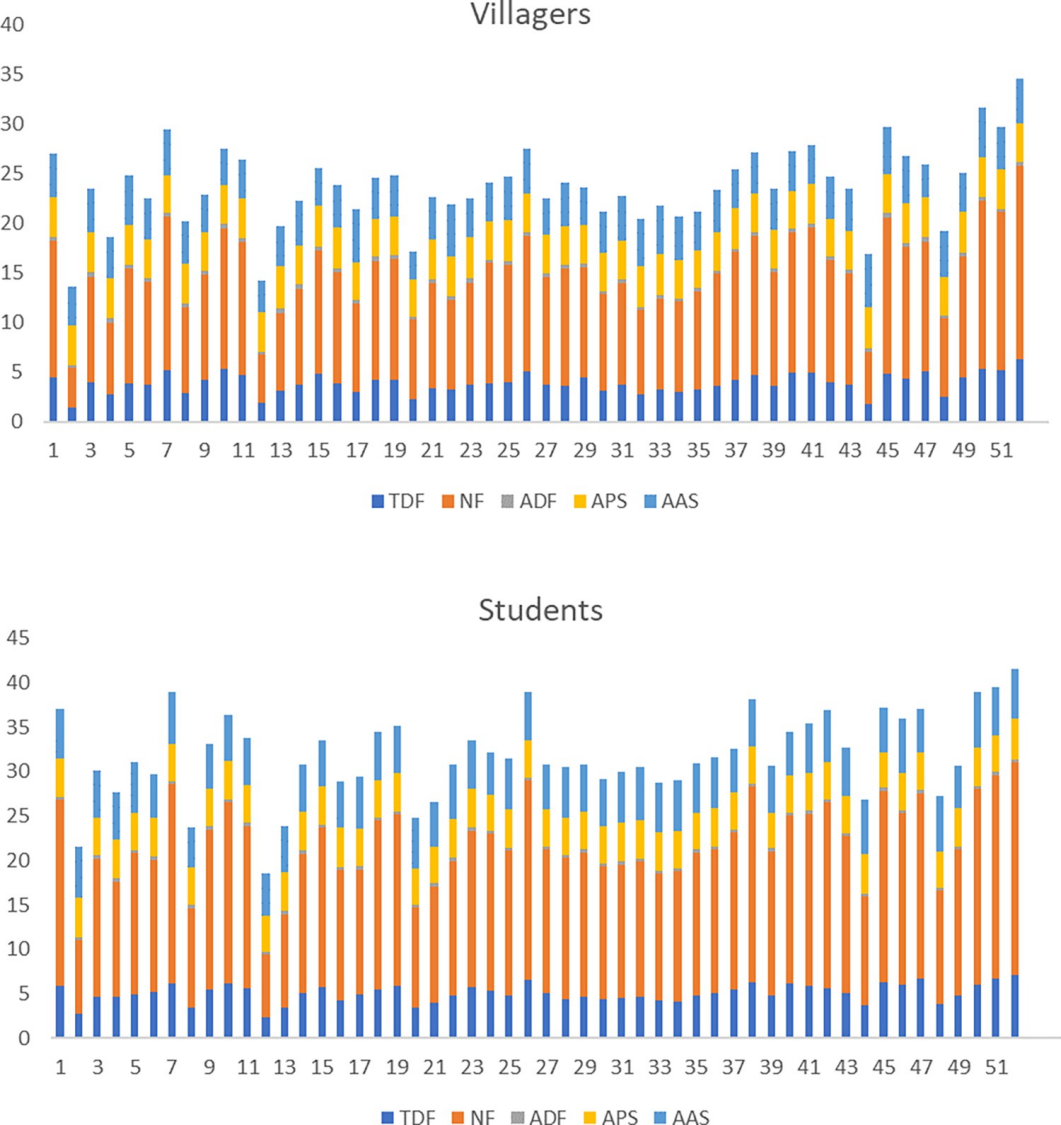
The mean value of eye movement indicators of villagers and students at species level. TDF: Total duration of fixations; NF: Number of fixations; ADF: Average duration of fixations; APS: Average pupil size; AAS: Average amplitude of saccades.1-52 respectively correspond to the species in Table 1.

In terms of species, there are significant differences between villagers and students in their total duration of fixations, number of fixations and average amplitude of saccades respectively (p<0.05), and the trends are basically the same. For the first two indicators, *Photinia×fraseri* has the highest value, *Metasequoia glyptostroboides*, *Photinia serratifolia*, *Koelreuteria bipinnata* and *Cunninghamia lanceolata* are also at the forefront. Meanwhile, villagers have higher values of the indicators on *Pinus thunbergii*, *Myrica rubra* and *Camellia japonica*, while students have higher values of the indicators on *Yulania biondii*, *Cercis chinensis* and *Hibiscus mutabilis*, and larger saccades on *Pinus massoniana*, *Acer buergerianum*, *Cunninghamia lanceolata*, *Jasminum mesnyi*, *Choerospondias axillaris*, *Camellia japonica*, and *Sapindus saponaria*. In addition, there are differences in average duration of fixations among villagers (p = 0.000<0.05) with higher values of *Chimonanthus praecox*, *Pinus thunbergii* and *Diospyros rhombifolia*, and differences in average pupil size among students (p = 0.000<0.05) with larger average pupil size on *Pinus massoniana*, *Photinia×fraseri* and *Malus halliana* (Fig 2).

**Fig 3:**
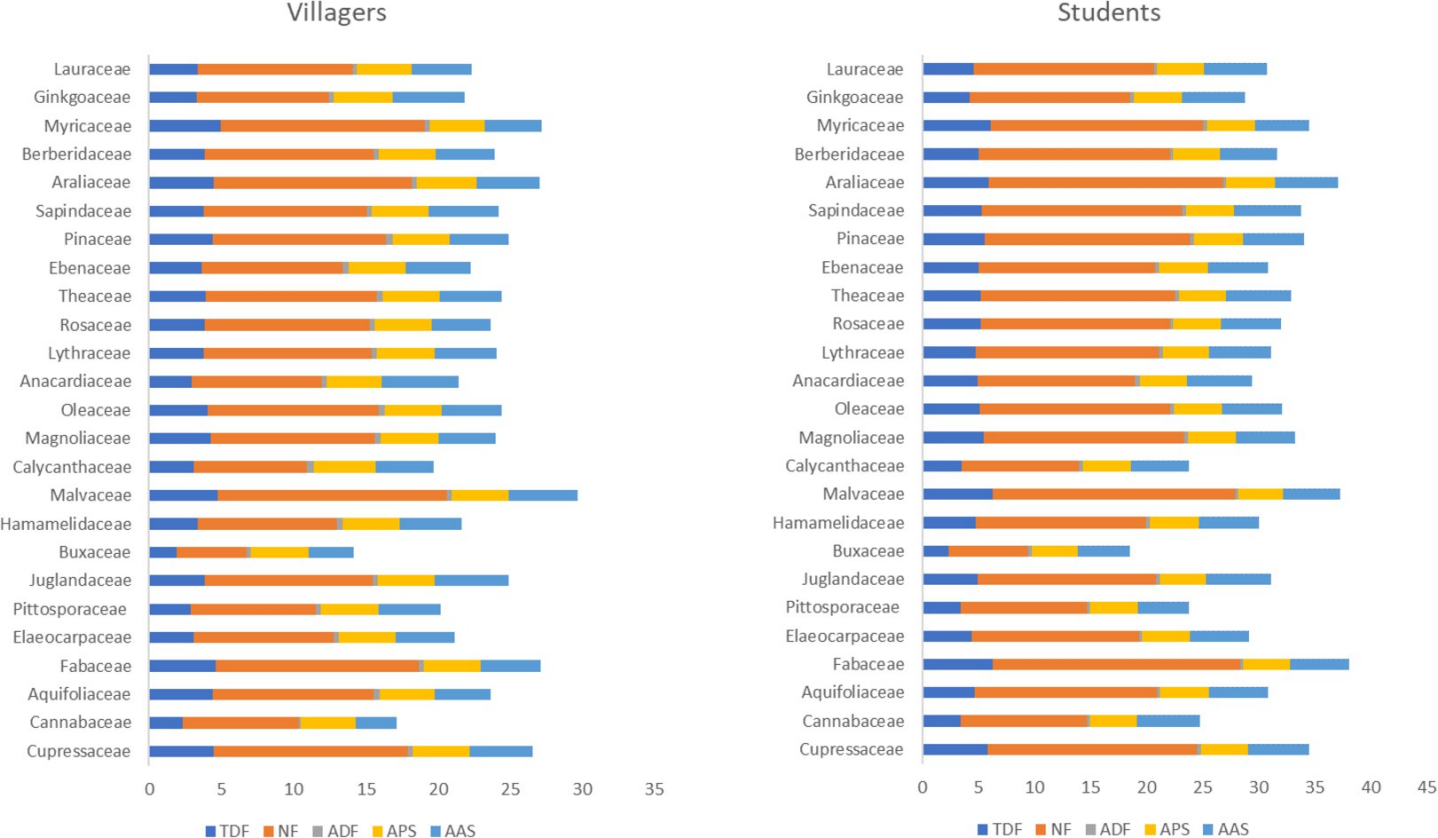
The average value of eye movement indicators of villagers and students at the level of families. TDF: Total duration of fixations; NF: Number of fixations; ADF: Average duration of fixations; APS: Average pupil size; AAS: Average amplitude of saccades.

Like species, there are significant differences in families in total duration of fixations, number of fixations and average amplitude of saccades between the two groups (p<0.05). *Fabaceae*, *Myricaceae*, *Malvaceae*, *Araliaceae*, *Cupressaceae*, *Pinaceae* and *Theaceae* are all in the forefront. Both groups have larger saccades in *Anacardiaceae*, *Juglandaceae*, *Ginkgoaceae*, *Sapindaceae*, *Cupressaceae*, *Araliaceae* and *Theaceae*. There are differences in average duration of fixations among villagers (p = 0.000<0.05), with higher values in *Calycanthaceae*, *Ebenaceae* and *Pinaceae*. There are also differences in the average pupil size of students (p = 0.014<0.05). The pupil sizes of *Pinaceae*, *Ebenaceae* and *Calycanthaceae* are larger (Fig 3).

#### Leaves

**Fig 4:**
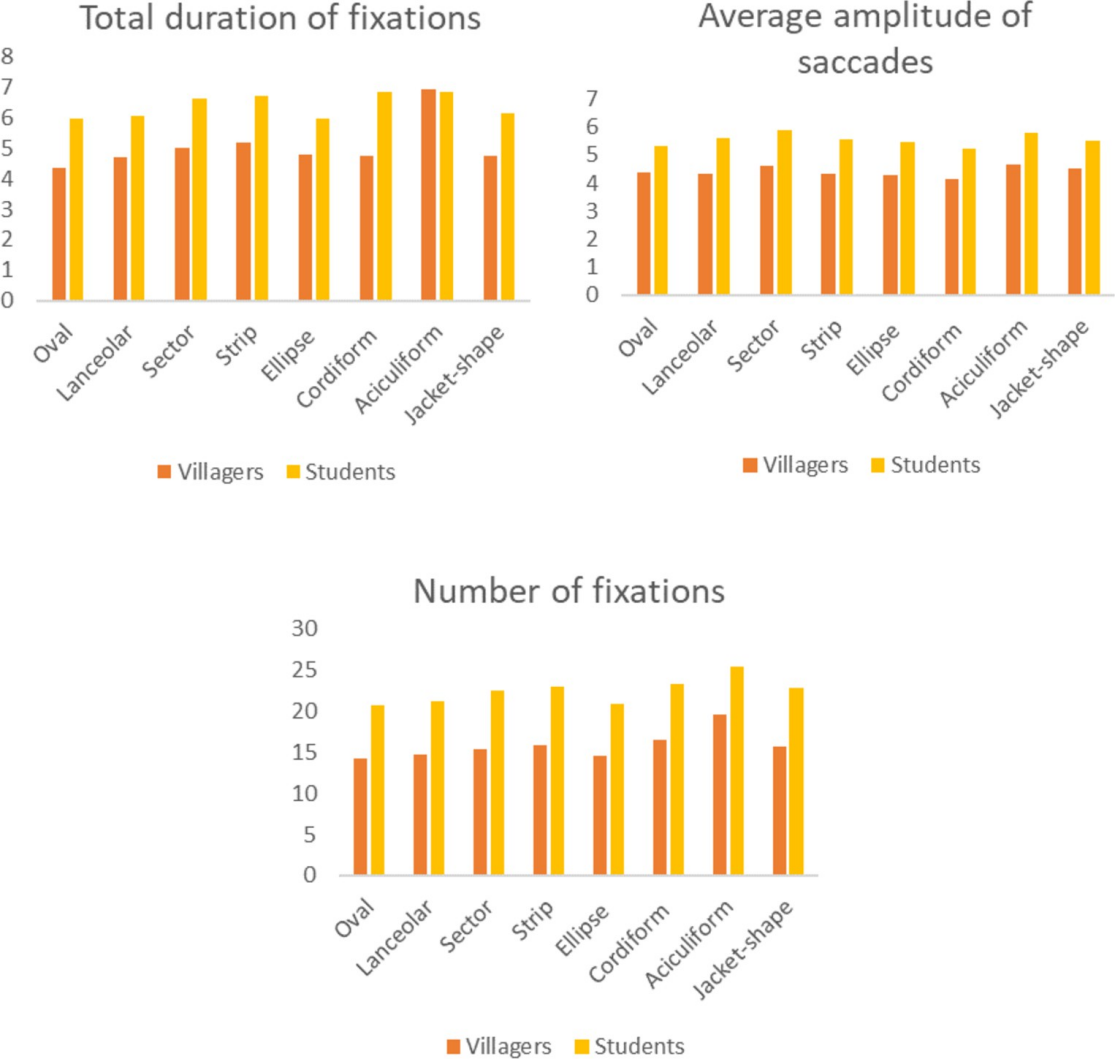
The average value of eye movement indicators of villagers and students with different leaf shapes.

**Fig 5:**
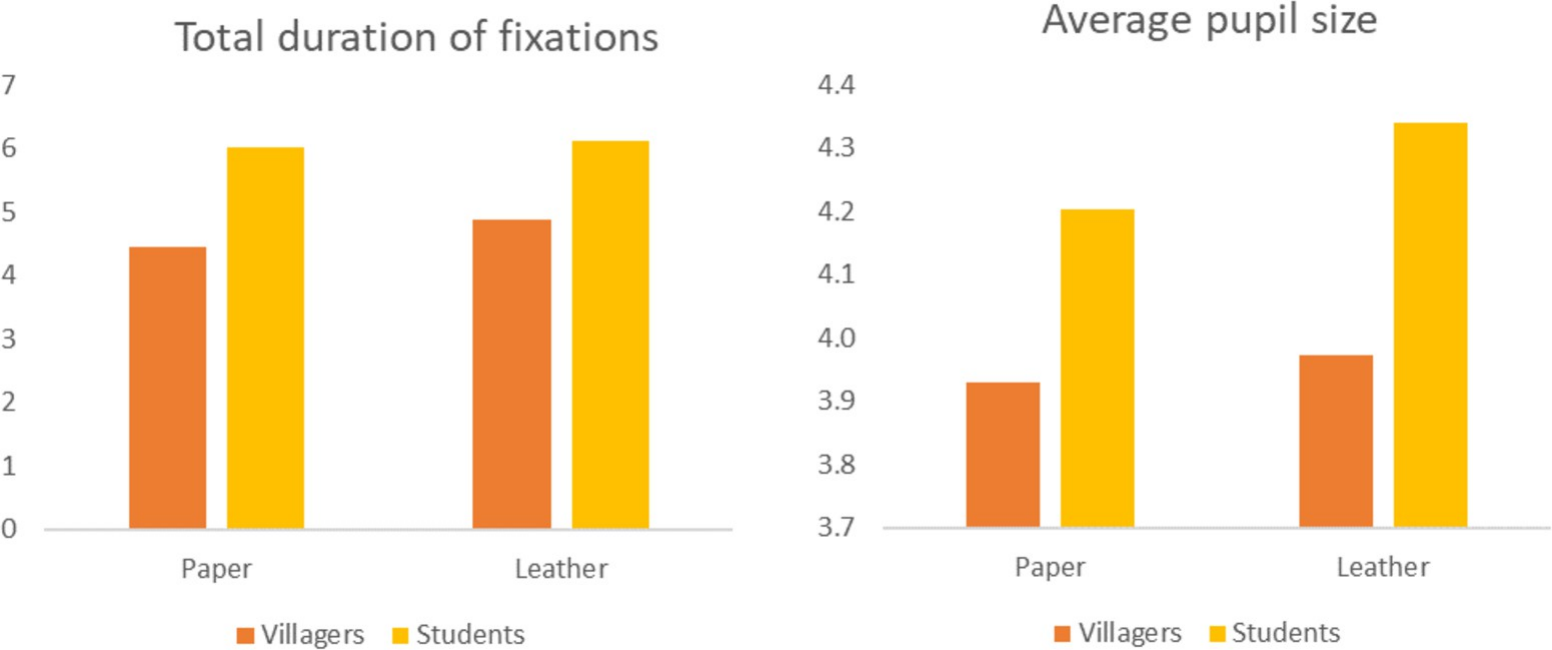
The average value of eye movement indicators of villagers and students on different leaf qualities.

There are significant differences in total duration of fixations and number of fixations between the two groups in different leaf shapes (p<0.05). The highest average value of the two indicators is aciculiform, followed by strip, cordiform and sector. The difference is that the average value of the student group is also higher in the jacket-shaped leaves. In addition, the average pupil size of different leaf shapes is different among students (p = 0.004<0.05), and the average values of aciculiform, strip and sector are higher still (Fig 4). In terms of leaf texture, there is a significant difference in the total duration of fixations of villagers (p = 0.004<0.05), and the average value of leather leaves is slightly higher than that of paper leaves. The average pupil size of students is different (p = 0.001<0.05), and the leathery leaves are higher than the paper leaves (Fig 5). There is also a significant difference among students in the average pupil size of leaves with or without leaf lobes (p = 0.027<0.05), and the results show that the average value of leaves without leaf lobes is higher.

#### Flowers

**Fig 6:**
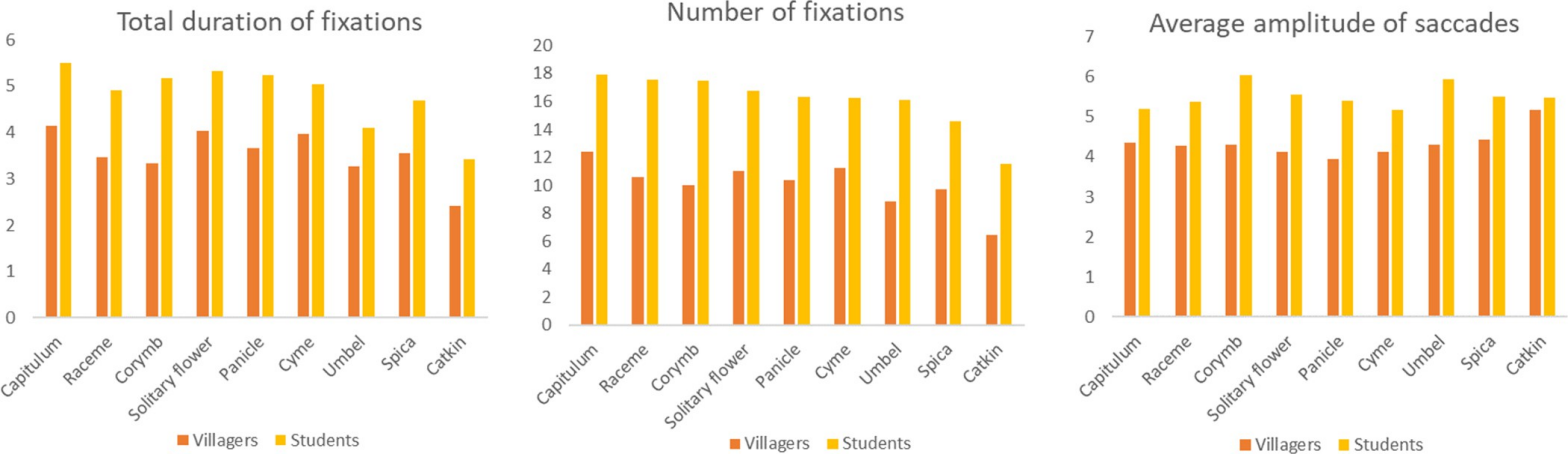
The average of eye movement indicators of different inflorescences by villagers and students.

**Fig 7:**
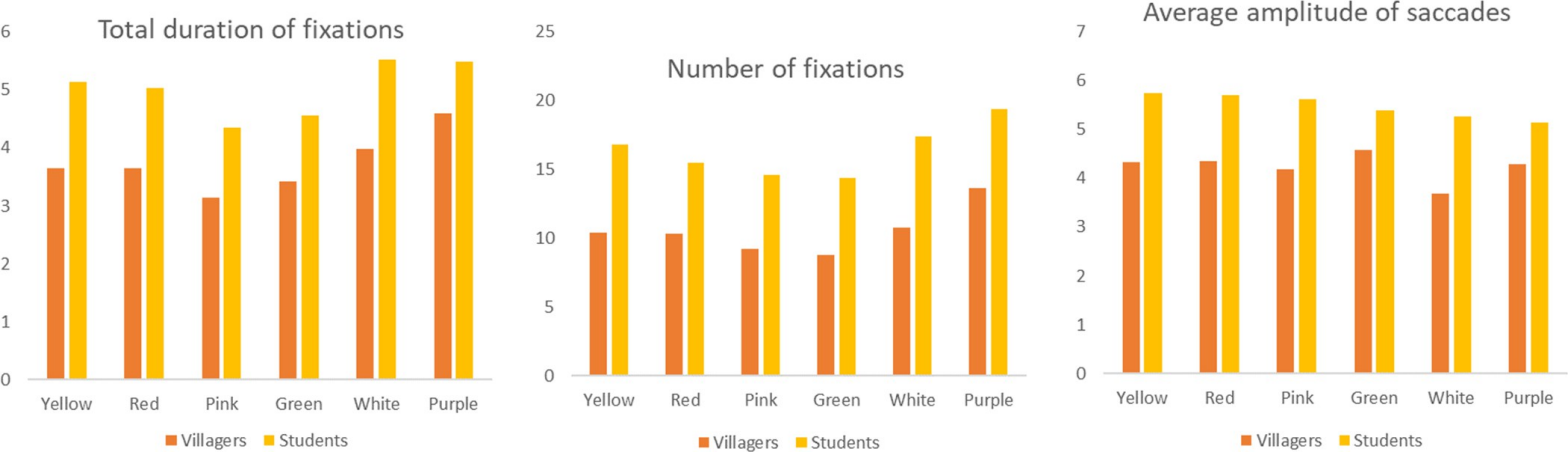
The average value of eye movement indicators of villagers and students for different colours.

There are significant differences between villagers and students in total duration of fixations and number of fixations for different inflorescences respectively (p<0.05), with the highest values of each indicator in capitulum, followed by solitary flower and panicle. The difference is that villagers have higher fixation time and number on cymes, while students have a higher number of fixations on corymb. The average amplitude of saccades of villagers is different (p = 0.039<0.05), with the saccade of corymb, umbel and solitary flower larger than that of other inflorescences. Correspondingly, villagers and students have the longest total fixation time when watching solitary flowers, followed by limited inflorescences and infinite inflorescences (Fig 6). There are differences between the two groups in the total duration of fixations and frequency, and the average amplitude of saccades of different colours respectively (p<0.05). The average arrangement of the first two indicators of the two groups is basically the same: purple is first, followed by white and yellow. But there are differences between the two groups in saccade amplitude. Except for yellow and red, the villagers have the largest saccade amplitude when they look at green, while the students have a larger saccade amplitude when they look at pink (Fig 7).

#### Fruits

On the whole, compared with students, the features of fruit have a greater influence on villagers. In terms of fruit size, there are significant differences among the five indicators of villagers (p<0.05). The total duration of fixations of medium fruit and micro fruit is the highest, while the average duration of fixations of large and medium fruit is longer, and the average pupil size and saccade amplitude of large and small fruit are larger. In the student group, there is significant difference (p<0.05) in other indicators except for the average duration of fixations. Similarly, the total duration of fixations and number of fixations of medium and micro fruit are at the forefront, and the average pupil size of large and small fruit is larger. But the difference is that the average eye saccade of students to small and micro fruit is larger.

**Fig 8:**
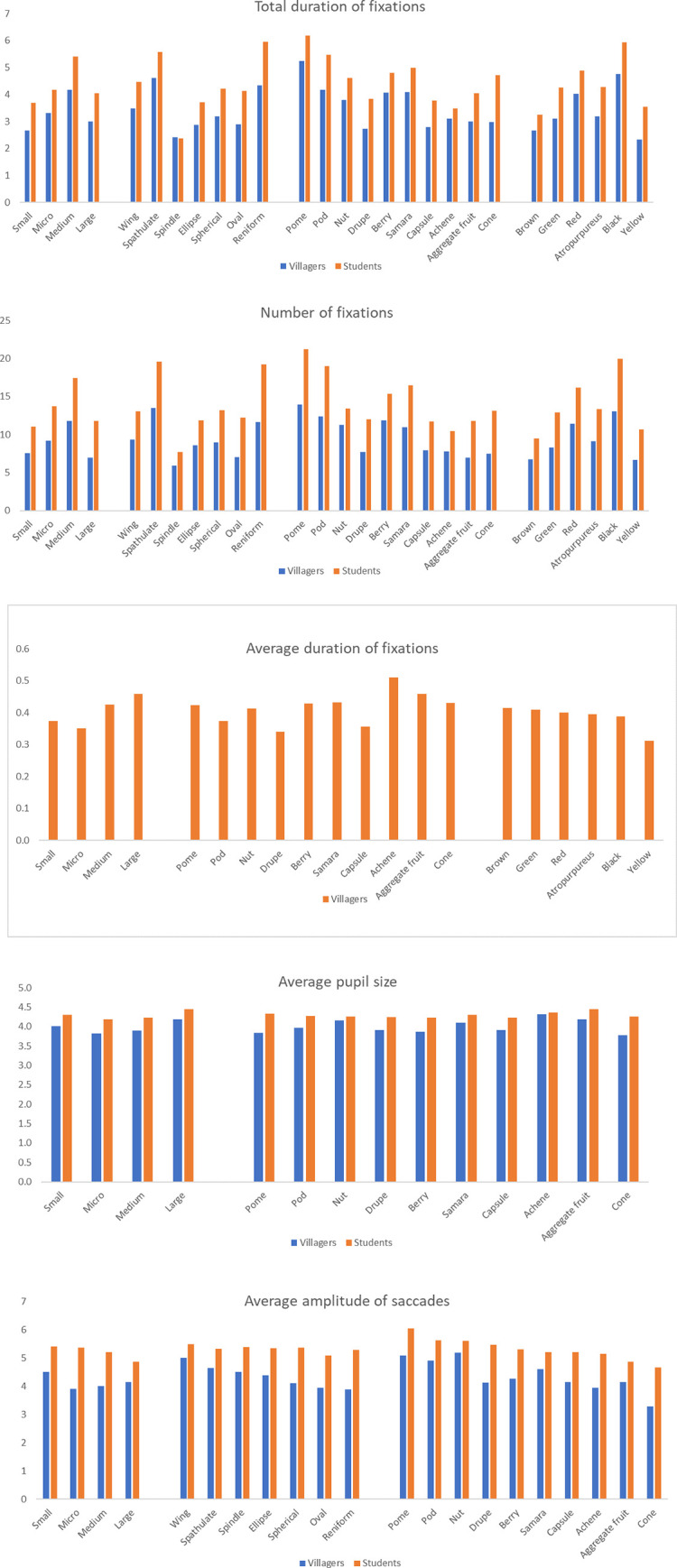
The mean value of eye movement indicators of villagers and students on the features of different fruits. Fruit sizes include small, micro, medium and large; Fruit shapes include wing, spathulate, spindle, ellipse, spherical et al. Fruits include pome, pod, nut, drupe, berry et al.; Fruit colours include brown, green, red, atropurpureus, black and yellow.

For fruit shapes, there are significant differences in total duration of fixations and number of fixations between the two groups (p<0.05). The values of spathulate and reniform are the highest, followed by the values of wing and spherical. There are also differences in the average amplitude of saccades of villagers (p = 0.001<0.05), and the saccade amplitude of wing, spathulate and spindle is larger. With regard to fruit types, there are differences in all the indicators of eye movement among villagers (p<0.05), but there is no difference in average duration of fixations and average pupil size among students (p>0.05). Villagers, as well as students, have the most total duration of fixations and number of fixations when watching pomes, pods, samaras and berries; the average duration of fixations and the pupil size of achene and aggregate fruit are higher; and the average eye saccade of nuts, pomes and pods is higher. In terms of colour, there are differences between villagers and students in average duration of fixations and frequency respectively(p<0.05). Both groups have the same average result of colour, with the highest values of black and red. There are also differences in average duration of fixations of villagers (p = 0.001<0.05), and brown and green have the longest fixation time (Fig 8).

### EEG data

The digital indicator eSense, which is calculated and processed by eSenseblem algorithm, represents people’s attention level and meditation level with specific values between 1 and 100. In this study, the values of the two indicators of the two groups are between 40 and 60, indicating that their indicators values are in the general range–that is, the baseline range value. According to the results of Mann-Whitney U-test, on the whole there are significant differences between villagers and students in two EEG indicators respectively (p<0.05), and students’ attention level is much higher than that of villagers, while their meditation degree is lower (Table 4).

**Table 4:** Mean value of EEG indicators and non-parametric test results of villagers and students as a whole.

Groups	Attention			Meditation		
	Mean	SD	p	Mean	SD	p
Villagers	46.48	4.895	0.000	56.56	4.645	0.038
Students	50.23	4.294		56.20	3.212	

#### Organs, species and families

On the two EEG indicators, the interaction effect between different living environments and different organ types, species and families are very significant (p<0.05). Separately, there are significant differences (p<0.05) between villagers and students in organ types, species and families, except that there is no difference in students’ meditation degree on different organs (p = 0.280>0.05).

**Fig 9:**
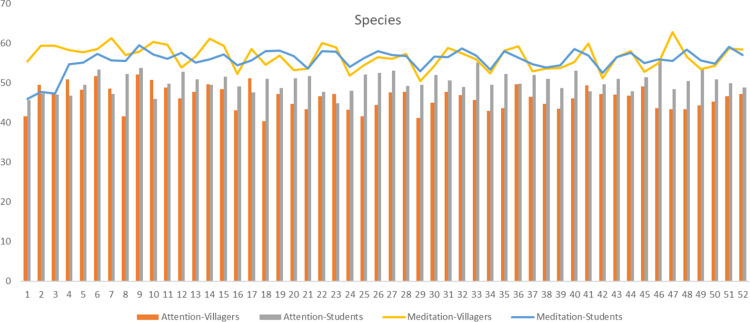
The average value of EEG indicators of villagers and students in different species. 1–52 respectively correspond to the species in Table 1.

The results of Kruskal-Wallis H-test show that villagers have the highest attention (M = 47.38) and meditation (M = 57.37) when watching flowers, followed by fruits and leaves, while students are the opposite: their highest attention lies in leaves (M = 50.68), followed by fruits and flowers. As to species, both villagers and students pay more attention to *Magnolia grandiflora* and *Fokienia hodginsii*, while villagers also pay more attention to *Choerospondias axillaris*, *Liquidambar formosana* and *Pinus thunbergii*, and students pay more attention to *Camellia japonica*, *Ginkgo biloba* and *Yulania liliiflora*. There is no overlap in meditation degree between the two groups. Villagers have a higher degree of meditation on *Yulania biondii*, *Koelreuteria bipinnata*, *Diospyros rhombifolia*, *Pinus thunbergii* and *Acer buergerianum*, while students have a higher degree of meditation on *Magnolia grandiflora*, *Metasequoia glyptostroboides*, *Sapindus saponaria*, *Myrica rubra* and *Jasminum mesnyi* (Fig 9).

**Fig 10:**
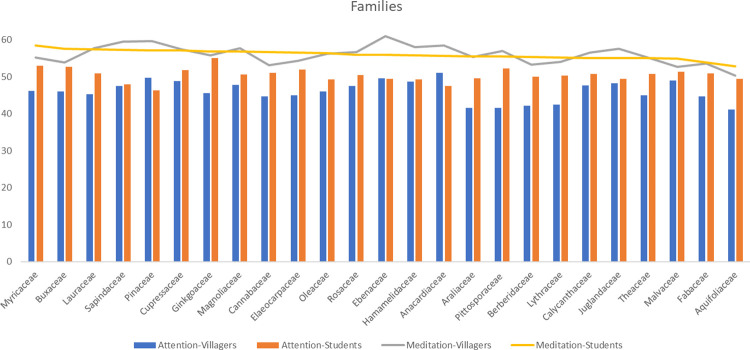
The average value of EEG indicators of villagers and students in different families.

For families, students have higher attention and meditation on *Myricaceae* and *Buxaceae*, with *Ginkgoaceae*, *Pittosporaceae* and *Elaeocarpaceae* also at the forefront, while villagers have the highest attention and meditation on *Anacardiaceae*, *Pinaceae* and *Ebenaceae* (Fig 10).

#### Leaves

**Fig 11:**
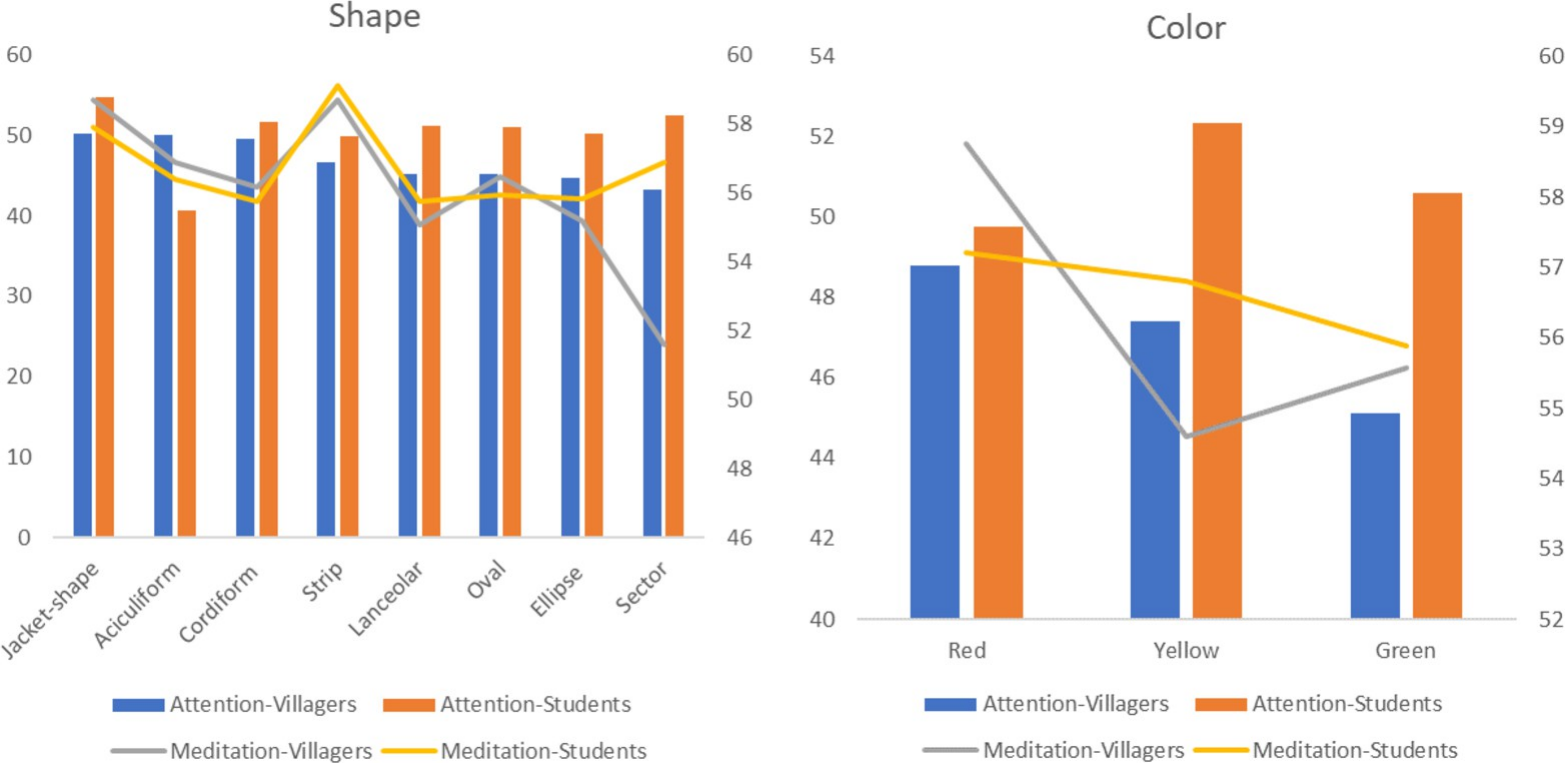
The average value of EEG indicators of villagers and students with different leaf shapes and colours.

Multivariate analysis of variance shows that there are differences (p<0.05) on attention in the interaction effects of different living environment with shape, colour and texture of leaves, but there are no differences in other features or in meditation. For leaf shape, there are significant differences (p<0.05) between villagers and students. Both groups have higher attention on jacket-shape and cordiform, and have the highest degree of meditation on jacket-shape and strip. Villagers also have higher attention on aciculiform and strip, while students have higher attention on sector and lanceolar. As to leaf colour, villagers have significant differences (p<0.05) in attention and meditation. Red leaves have the highest mean value, followed by yellow and green leaves. Students only have differences (p = 0.015<0.05) in attention, which are followed by yellow, green and red. On leaf texture, there is a significant difference (p = 0.004<0.05) in students’ attention. The paper leaf (M = 51.45) is higher than the leather leaf (M = 50.30), contrary to the results for the villagers. But there is no difference (p = 0.566>0.05) between the two leaf textures. There is a significant difference (p = 0.017<0.05) in villagers’ attention on leaf lobes. The average value of leaves with leaf lobes (M = 47.05) is higher than that of leaves without leaf lobes (M = 45.30), which is consistent with students, but there is no difference (p = 0.260>0.05) in students’ attention (Fig 11).

#### Flowers

**Fig 12:**
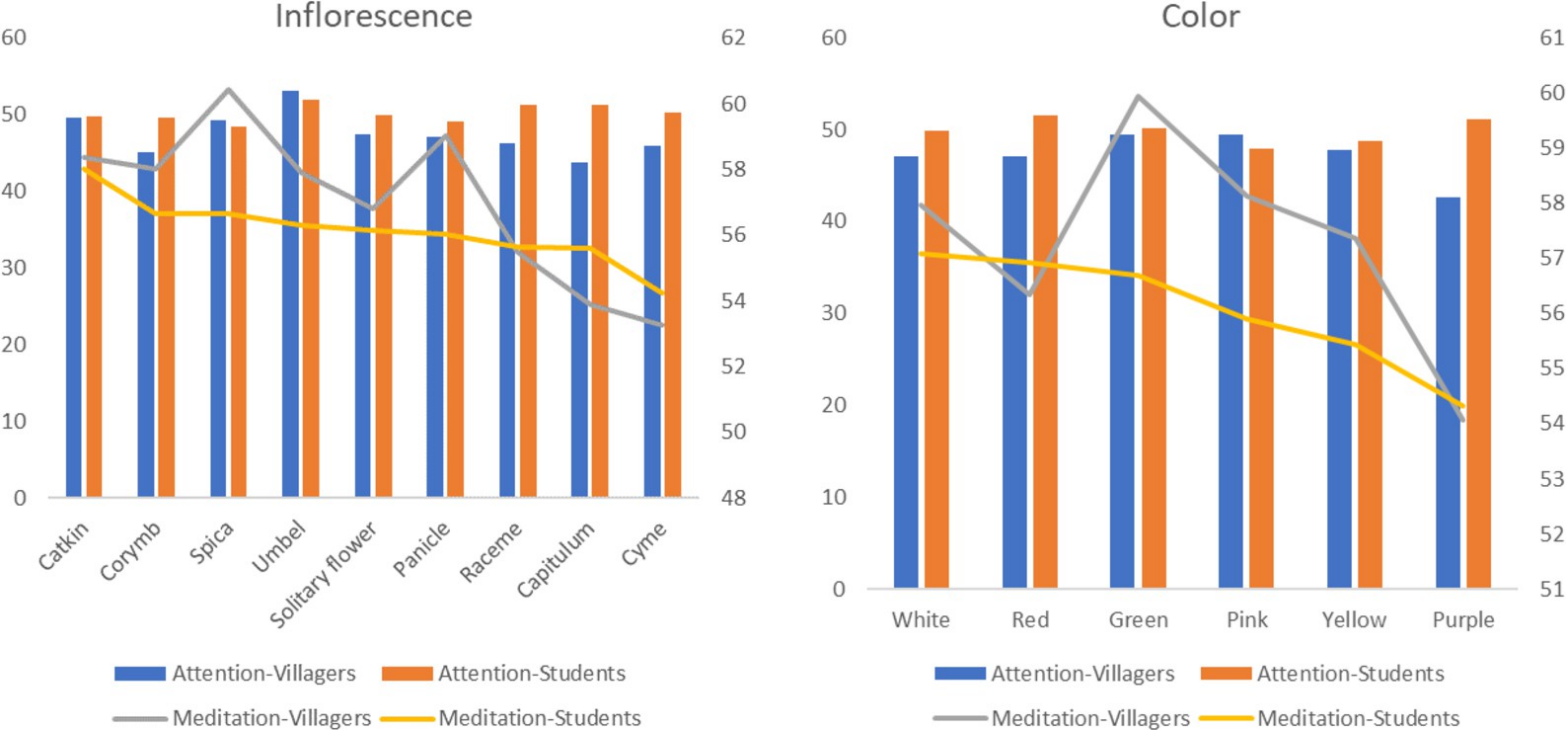
The average value of EEG indicators of villagers and students on different inflorescences and flower colours.

The interaction effect between different living environment and all features of flowers with regard to attention and meditation is significant (p = 0.000<0.05). In different inflorescences, there are significant differences between villagers and students in the two indicators respectively (p<0.05). Both groups have the highest attention on umbels, and the villagers have higher average values for catkins, spicas and solitary flowers, while students have higher attention on racemes, capitulums and cymes. In terms of meditation degree, the average value is higher in spica and catkin. Villagers and students also have a high degree of meditation in panicles and corymbs respectively. Furthermore, for the inflorescence types, there is only a significant difference in meditation degree between the two populations (p<0.05), and the infinite inflorescence has the highest value, followed by solitary flower and limited inflorescence. With regard to flower colours, there are significant differences between villagers and students in the two indicators respectively (p<0.05). Villagers are more focused on pink, green and yellow, while the students are more focused on red, purple and green. In meditation, the villagers have the highest values of green, pink and white, while students have the highest values for white, red and green in turn (Fig 12).

#### Fruit

**Fig 13:**
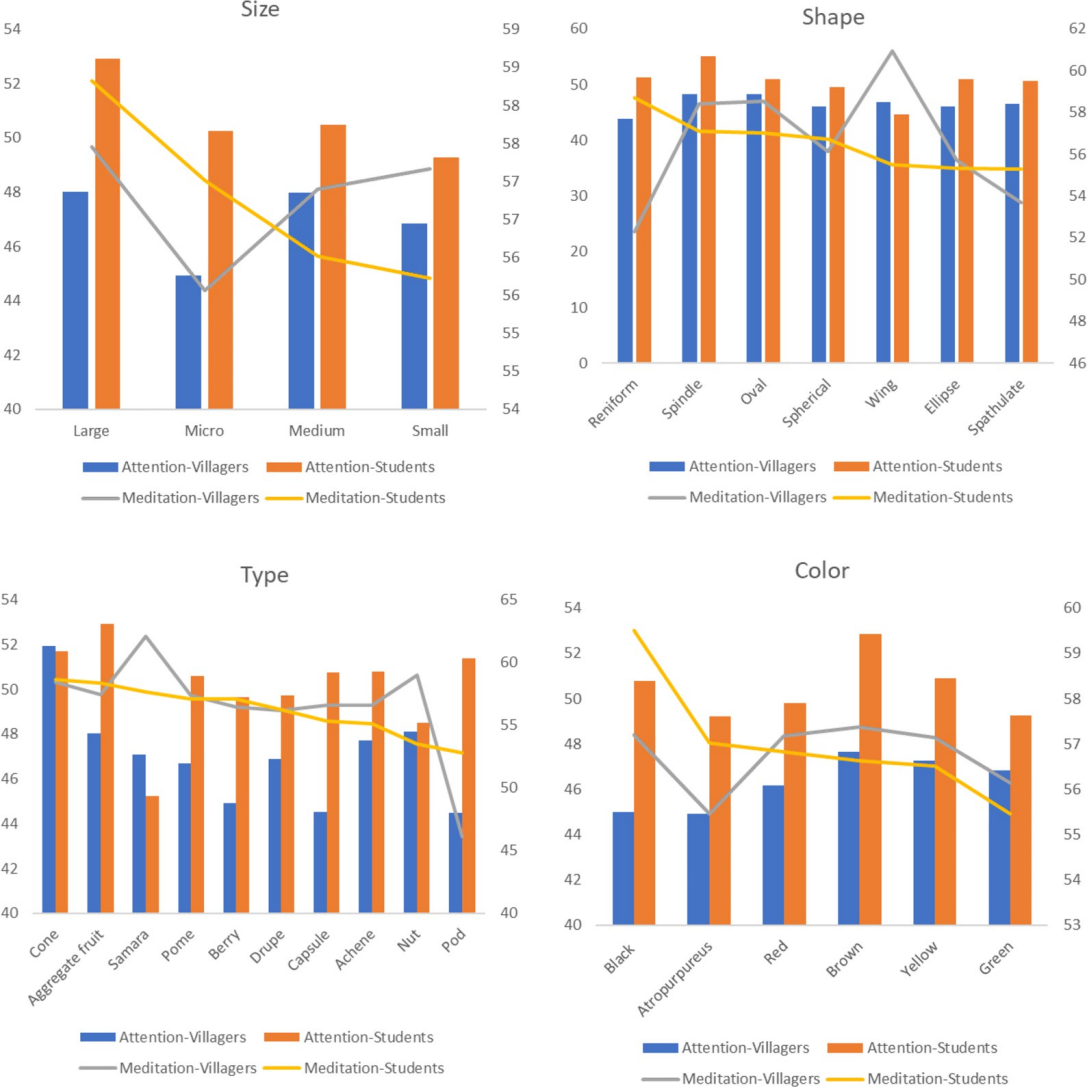
The average value of EEG indicators of different fruit features between villagers and students.

Except for fruit colour, the interaction effect between different living environment and other features of fruit is very significant (p<0.05), and there are significant differences (p<0.05) between villagers and students in most features respectively. In terms of fruit size, large fruit has the largest average value of the two indicators among the two groups. In terms of attention, villagers pay more attention to medium fruit, followed by small and micro fruits, and students pay more attention to medium, micro and small fruits. In terms of meditation degree, villagers are highest on small fruit, followed by medium and micro fruits, while students are highest on micro, medium and small fruits. With regard to fruit shapes, villagers and students are more focused and relaxed on oval and spindle; third is wing shape for villagers and reniform for students. For fruit types, villagers pay higher attention to cones, nuts and aggregate fruits, and the meditation degree of samaras is the highest. Students have the highest values of aggregate fruits and cones, and their attention and meditation degree of pods and samaras are also at the forefront. In terms of fruit colours, there is no difference in the meditation degree of villagers, but they focus more on brown, yellow and green. Students are similar to villagers, focusing more on brown, yellow and black. In meditation degree, the values of brown, black and red are higher for students (Fig 13).

### Correlation analysis

The Pearson correlation analysis between the five eye movement indicators and the two EEG indicators showed that there was a strong positive correlation (p = 0.000, Pr = 0.96) between total duration of fixations and number of fixations, a significant weak negative correlation (p = 0.015, Pr = –0.221) between average pupil size and attention, and a significant weak positive correlation(p = 0.028, Pr = 0.200) between average duration of fixations and meditation degree.

## Discussion

### Visual preference and difference through eye movement

There is no significant interaction between living environment and local plant features in indicators of eye movement–that is, living environment will not affect people’s eye movement features when watching different plant pictures, which is consistent with the research results of Luo et al. [30], who points out that the aesthetic preferences of Xiamen local high school students and Xinjiang high school students in Xiamen for new and old landscapes tend to be consistent. Besides living environment, Paraskevopoulou et al. [23] used eye tracking technology to study the influence of seasonal colour changes of plants on patients with mental disorders, and also found that age and gender did not affect the preferences of participants. We believe it may be difficult to discover the specific impact of the environment on people only by using eye movement indicators.

People prefer attractive stimuli, which lead to a longer fixation time and a larger number of fixations [79]. At the same time, this study found that the two indicators have a strong positive correlation, so they can jointly reflect the same phenomenon: the preference level of participants [80]. As for species, *Photinia×fraseri*, *Metasequoia glyptostroboides*, *Photinia serratifolia*, *Koelreuteria bipinnata* and *Cunninghamia lanceolata* have higher visual appeal, while for families, both groups prefer *Fabaceae*, *Myricaceae*, *Malvaceae*, *Araliaceae*, *Cupressaceae*, *Pinaceae* and *Theaceae*. In terms of organ types, both groups are most easily attracted by leaves, followed by flowers and fruits. This is different from the research results of Rahnema et al. [19] and Yin et al. [81], which indicate that flowers are more attractive than leaves because the visual attention of participants is driven by many different factors that have nothing to do with subjective and declarative preferences [41]. For example, Lindemann-Matthies and Bose [82] found that the participants indicated in the questionnaire that they liked plants with large or colourful flowers, but when they were asked to choose 25 kinds of local wild plants from 779 kinds to create their favorite lawn patch, only one-third of the flowering plants were selected. Among the different features of leaves, aciculiform, strip, cordiform and sector shapes have higher preference rates. Lindemann-Matthies and Bose [82] also point out that leaf shape has a significant impact on people’s preferences. Villagers prefer leather leaves a with stiff texture and strong lustre, while students have no obvious preference between leather leaves and paper leaves. Among the different features of flowers, capitulums, solitary flowers and panicles attract the attention of both groups. Furthermore, they both prefer solitary flowers, followed by finite inflorescences and infinite inflorescences. In terms of flower colours, purple, white, yellow and red have higher preference rates. This discovery supports the results of other studies [19], which show that red, purple and yellow have the highest preference rates. Among the different types of fruits, the two groups prefer medium and micro fruits, and spathulate, reniform, wing and spherical fruits have higher visual appeal. Compared with ellipse and oval shapes, they are more prominent and unique in shape, and tend to arouse curiosity, which impacts visual appeal [41]. Fruit types such as pomes, pods, samaras and berries have a higher preference rate, and black and red colours are attractive to the two groups. Red can promote people’s optimistic mood, while dark colours can produce a relaxed mood [83].

On the whole, students’ average duration of fixation is shorter than that of villagers, and they can grasp the information displayed by the pictures more quickly, which may be influenced by their educational background [27, 47]. There is no difference in species and families among students, but *Chimonanthus praecox*, *Pinus thunbergii*, *Diospyros rhombifolia*, *Calycanthaceae*, *Ebenaceae* and *Pinaceae* show a lot of information to villagers, and they need to spend more time to understand it. In terms of plant organs, although leaves have a high preference, the most prominent feature of plants is the differentiation of flowers and fruits in different seasons [16], so the various features of leaves will not affect the processing time of the information they convey, and require considerable time. However, while the features of fruit convey much information, this only affects villagers, to whom large and medium fruits convey the most information, and achene and aggregate fruits need more time to analyse, as brown and green fruits do.

The average pupil size of students is larger than that of villagers as a whole, indicating that students’ visual response to stimulation is stronger. Different features of leaves have different effects on students’ visual stimulation, and they are more sensitive to aciculiform, leather leaves and leaves without leaf lobes. Various features of fruit have a greater influence on villagers. Achene and aggregate fruits are highly stimulating and, like students, villagers are sensitive to large and small fruits.

The average amplitude of students’ saccade is much larger than that of villagers–that is, students can find and lock the AOI more easily, and have a more sensitive response to the stimulation of plants, which corresponds to the difference between experts and non-experts discussed by Pihel et al. [47]. Specifically, there are obvious differences among villagers’ eye saccade. In terms of flowers, corymbs, umbels and solitary flowers are more obvious to them. Yellow and red are more likely to attract the attention of the two groups, because red can stimulate and distract visual attention [83], while yellow flowers can be used to create pleasant places [84]. In terms of fruit, the features of wing, spathulate and spindle shapes are obvious to villagers. At the same time, like students, nuts, pomes and pods are more prominent.

### Physiological characteristics and differences through EEG

We found a weak negative correlation between average pupil size and attention: when the participants are stimulated by vision, the pupil response increases [85], while the mental attention level decreases to some extent. This is different from the research results of some scholars [57]. The results also show a weak positive correlation between the average duration of fixations and the meditation value, which means that when the participants need more time to receive and process a large amount of information transmitted by experimental stimulus materials, their average duration of fixations will increase [76], while the visual stimulus will decrease and the meditation value will increase. It is understandable that the correlation between eye movement indicators and EEG indicators is weak. Garcia-Madariaga et al. [86] mentions that no connection exists between explicit preference measured by eye tracking and attention measured by EEG, while Vettori et al. [87] reached a completely opposite conclusion, pointing out that eye tracking and EEG measurement are strongly correlated. Therefore, the correlation between them needs further research.

On the whole, students’ mental attention level when watching plant stimulation is much higher than that of villagers, which may be due to the influence of educational background [27]. Students might do some plant recognition activities during watching, while villagers simply browse the pictures. Accordingly, students are engaged in more brain activities, and the mental meditation degree is lower than that of villagers. There is a very significant interaction effect between different living environment and various native plant features in EEG indicators. It can be seen from the visual indicators alone that there is no difference between students and villagers when watching plant pictures, and the addition of EEG further supplements and reveals the real situation [76]. Therefore, there are in fact very different physiological reactions between the two groups when watching. This is similar to the findings of other scholars. In the eye tracking data, there is no interaction between the group and the stimulus types, but the EEG indicators are the opposite, and the difference between the two groups can clearly be seen [87]. As to organ types, when villagers watch flowers, the two EEG indicators are the highest, followed by fruit and leaves, while students have completely different attention. For leaf colours, red leaves can increase villagers’ attention and meditation, and green leaves have the lowest corresponding value, but green leaves can improve students’ attention, while red leaves have the lowest effect. In terms of flowers, besides umbels, catkins, spicas and solitary flowers also can promote villagers’ attention, while racemes, capitulums and cymes can attract students’ attention. In this sense, there is a significant difference between these two groups. It can be seen that obvious features and bright colours can relieve the spirit of villagers [75], and improve their attention and meditation, such as flowers with catkins or spicas and red leaves. On the contrary, relatively less prominent plant features and simpler structure, such as large areas of green leaves and flowers with racemes and capitulum, can increase students’ EEG values.

This study introduced two physiological technologies, eye tracking and EEG, which transcended the limitations of the previous studies only from the perspective of ecology or using subjective measurement methods. At the same time, it was found that different environments would have certain influences on people’s viewing of plants. These not only provide an objective technical support for the selection of suitable plants in different landscape construction, which makes the plant selection more rule-based and can even enable generalization, but also provide a new research perspective for landscape preference-related fields.

### Limitations and prospects

However, this study has some limitations. First, we have not completely covered all the features of plant organs in the study. Future studies need to further improve the selection of experimental plants, in order to achieve full coverage of features as much as possible. On this basis, it is necessary to use plant individuals, populations or communities as experimental stimulation materials to make research closer to people’s natural observation habits. At the same time, it is necessary to enrich more group samples in different living environments, to provide scientific suggestions for the use of plants in the process of creating more different landscapes. In addition, the EEG detection instrument used in this study is portable and simple, and its accuracy and synchronization with eye tracking need to be improved. More advanced EEG data instruments should be used, which can monitor 14, 64 or 128 channels, and can be directly connected with the eye tracker to synchronize more accurately in further research.

## Conclusion

In this study, villagers and students were taken as research groups, and their preferences and differences on plant organ features were explored by using eye tracking and EEG technology. According to the eye movement indicators, there is no obvious difference in preferences between villagers and students. Both groups prefer leaves, especially aciculiform and leather leaves, followed by flowers and fruits. Solitary flowers, capitulums and purple flowers have the highest preference rates, and medium-sized fruits with diameters of 3–5 centimeters, spathulate, black and pome fruits have the highest visual appeal. However, the application of EEG technology further reveals significant physiological and psychological differences between villagers and students when they watch the stimuli. On the whole, students’ attention is much higher than that of villagers, while their meditation degree is the opposite. This study has certain significance for plant selection in landscape construction in rural areas and campuses in China. We think *Photinia×fraseri*, *Metasequoia glyptostroboides*, *Photinia serratifolia*, *Koelreuteria bipinnata* and *Cunninghamia lanceolata* are the preferred plants in both environments. For rural areas, *Pinus thunbergii*, *Myrica rubra* and *Camellia japonica* are also highly recommended, and plants with obvious features and bright colours are also suitable for rural landscape construction. On campus, *Yulania biondii*, *Cercis chinensis* and *Hibiscus mutabilis* are recommended species, and plants without too-prominent characteristics and with simple structures are also suitable for campus landscape construction.
